# Current Status of Rift Valley Fever Vaccine Development

**DOI:** 10.3390/vaccines5030029

**Published:** 2017-09-19

**Authors:** Bonto Faburay, Angelle Desiree LaBeaud, D. Scott McVey, William C. Wilson, Juergen A. Richt

**Affiliations:** 1 Department of Diagnostic Medicine and Pathobiology, College of Veterinary Medicine, Kansas State University, Manhattan, KS 66506, USA; 2 Center of Excellence for Emerging and Zoonotic Animal Diseases (CEEZAD), Department of Diagnostic Medicine/Pathobiology, College of Veterinary Medicine, Kansas State University, Manhattan, KS 66506, USA; 3 Department of Pediatrics, Stanford University School of Medicine, Stanford, CA 94305, USA; dlabeaud@stanford.edu; 4 United States Department of Agriculture, Agricultural Research Service, Arthropod-Borne Animal Disease Research Unit, Manhattan, KS 66506, USA; Scott.McVey@ARS.USDA.GOV (D.S.M.), William.Wilson@ars.usda.gov (W.C.W.)

**Keywords:** Rift Valley fever virus, vaccines, livestock vaccination

## Abstract

Rift Valley Fever (RVF) is a mosquito-borne zoonotic disease that presents a substantial threat to human and public health. It is caused by Rift Valley fever phlebovirus (RVFV), which belongs to the genus Phlebovirus and the family Phenuiviridae within the order Bunyavirales. The wide distribution of competent vectors in non-endemic areas coupled with global climate change poses a significant threat of the transboundary spread of RVFV. In the last decade, an improved understanding of the molecular biology of RVFV has facilitated significant progress in the development of novel vaccines, including DIVA (differentiating infected from vaccinated animals) vaccines. Despite these advances, there is no fully licensed vaccine for veterinary or human use available in non-endemic countries, whereas in endemic countries, there is no clear policy or practice of routine/strategic livestock vaccinations as a preventive or mitigating strategy against potential RVF disease outbreaks. The purpose of this review was to provide an update on the status of RVF vaccine development and provide perspectives on the best strategies for disease control. Herein, we argue that the routine or strategic vaccination of livestock could be the best control approach for preventing the outbreak and spread of future disease.

## 1. Introduction

Rift Valley fever (RVF) is a mosquito-borne zoonotic disease that was first recognized as an acute and highly fatal disease of lambs associated with heavy rains and accompanied by illness in humans in the early twentieth century in the Rift Valley region in Kenya [1]. However, it was not until 1930 when the causative agent, Rift Valley fever phlebovirus (RVFV), was first isolated [2]. Since then, major outbreaks have occurred affecting livestock and humans in several countries in Africa, including Egypt, Kenya, South Africa, Madagascar, Mauritania, Senegal, and The Gambia [3,4,5,6]. In 2000 and 2001, a major outbreak of RVFV inflicting severe disease and economic loss was first reported outside the African continent in Saudi Arabia and Yemen in the Arabian Peninsula [7]. Rift Valley Fever (RVF) disease is most severe in sheep, cattle, and goats, resulting in high mortality in new born animals and mass abortion upon infecting pregnant animals [8,9,10]. Human infections are typically characterized by self-limiting febrile illness but may occasionally, in about 1 to 2% of affected individuals, progress to more severe disease that includes meningoencephalitis, retinitis, fulminant hepatitis, blindness, or a hemorrhagic syndrome with a case fatality rate of 20–50% [11,12].

**Figure 1:**
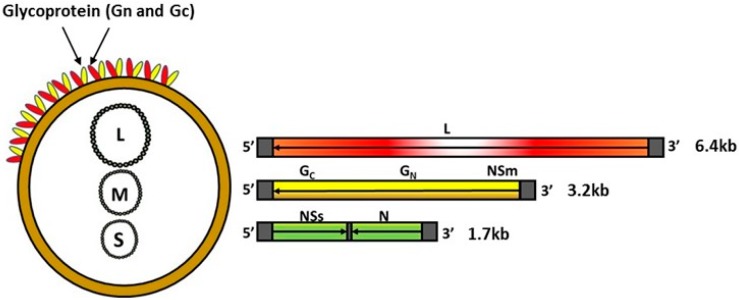
Schematic illustration of RVFV genome organization. Left panel shows the virion containing small (S), medium (M), and large (L) RNA segments; the panel also shows the surface glycoproteins Gn and Gc incorporated into the envelope lipid bilayer. Left panel shows the schematic representation of the RNA segments and the coding strategy. L = L protein; NSm = non-structural protein M; glycoproteins Gn, Gc; N = nucleoprotein; NSs = non-structural protein S. The arrows indicate the coding strategy of the virus.

RVFV belongs to the genus Phlebovirus within the family Phenuiviridae of the order Bunyavirales and contains a single-stranded, negative-sense, segmented RNA genome (Figure 1). The genome encodes four structural proteins, the nucleoprotein (N), the glycoproteins Gn and Gc, and the L polymerase; two non-structural proteins, NSs and NSm; and a 78-kDa protein of an unknown function [13,14,15]. Although both NSm and NSs proteins play a critical role in viral pathogenesis—NSm serving as an anti-apoptotic protein [14], and NSs a major viral virulence factor that inhibits host innate immune responses [16,17,18,19,20,21], they are dispensable for RVFV replication in vitro and in vivo [15,22,23]. The surface glycoproteins Gn and Gc play a role in virus attachment to initiate infection and have been shown to carry virus neutralizing epitopes [24,25,26]. Thus, the surface glycoproteins Gn and Gc have been the main targets for the development of novel RVF vaccines. Additionally, advances in knowledge relating to the molecular biology of the RVFV agent has been utilized by various research groups to develop DIVA—compatible vaccines [23,27,28,29].

RVFV belongs to the World Organization for Animal Health (OIE) list of notifiable diseases [30], and is classified as an overlap select agent by the Centers for Disease Control and Prevention (CDC) and United States Department of Agriculture (USDA). The virus has great potential for transboundary spreading. The wide distribution of competent vectors in non-endemic areas [31,32,33,34,35] coupled with the effects of climate change [36] and potential use for bioterrorism emphasizes the need for safe and efficacious vaccines. There is no licensed or commercially available vaccine for human use; and while a variety of vaccines for livestock have been developed, there are no fully licensed RVFV vaccines approved for veterinary use outside endemic areas [8,37,38,39,40,41,42,43,44]. In the past decade, various strategies to develop efficacious RVFV vaccines have been undertaken by several research laboratories or groups. These have included subunit vaccines [28,45,46], DNA vaccines [47,48], virus-like particles (VLPs) [29,46,49,50,51], virus replicon particle vaccines [52,53,54], virus-vectored vaccines [55,56,57,58], modified live vaccines (developed from recombinant viruses engineered using reverse genetics) [22,23,59,60,61], live attenuated vaccines [62,63,64,65,66,67], and inactivated whole virus vaccines [68]. These vaccines have been used and/or evaluated in different animal models such as mice, sheep, cattle, and non-human primates, as well as in human volunteers. To facilitate RVF vaccine development, various animal models have been developed (reviewed in Ross et al. [69]), including the development of ruminant models [70,71,72] for the evaluation of a novel subunit vaccine [28]. The purpose of this review was to provide an up-date on the status of RVF vaccine development and provide perspectives on the best strategies for disease control in endemic and non-endemic countries.

## 2. Vaccines

In endemic and non-endemic areas, vaccinating livestock against RVFV represents the most sustainable strategy to mitigate the impact of RVF on livestock agriculture. The earliest vaccines such as inactivated vaccines [73] and the live-attenuated Smithburn vaccine [66] were developed from virulent RVFV isolates using conventional technologies. Although these vaccines have contributed significantly to the control of RVF in endemic countries in Africa, their production and use has been associated with a certain level of risk, and they lack important attributes, such as the ability to DIVA, for use in non-endemic countries. In recent years, several research groups or laboratories have reported substantial progress in the development of novel vaccines. Many of the achievements have resulted from collaborations between human and veterinary research groups, thus presenting a classic “One Health” approach to combating RVF as a major zoonotic disease threat to both human and animal health. While many of the vaccines were initially tested in mice in proof-of-concept studies, several have progressed to evaluation in the natural target host, sheep or cattle, or have met the criterion of DIVA (Table 1). In this review, we have broadly classified RVF vaccines into: 1. Conventional vaccines (referring to vaccines produced using non-recombinant DNA technology methods), and 2. Novel vaccines (referring to vaccines produced using recombinant-nucleic acid technology adapted from the classification by USDA) [74]. Novel vaccines are further categorized into: (i) Type I vaccines (composed of antigens produced by recombinant nucleic acid technology); (ii) Type II vaccines (consisting of genetically attenuated viruses created by deletion of genes encoding virulence factors or proteins dispensable for virus replication); and (iii) Type III vaccines (consisting of live viruses into which DNA encoding protective antigens are introduced (virus-vectored vaccines)).

## 3. Conventional RVF Vaccines

### 3.1. Formalin-Inactivated

The initial application of RVF vaccines started with inactivated vaccines [10]. The RVFV Entebbe strain, isolated from a mosquito in Uganda, was the first virus strain used as a vaccine after being formalin-inactivated. A formalin-inactivated vaccine based on the Entebbe virus, named NDBR103, was manufactured using a mouse master seed that had undergone 176 intraperitoneal or intravenous passages in mice followed by amplification in African Green Monkey cells [75,96]. In 1977 and thereafter, the NDBR103 vaccine was used to vaccinate 500 human volunteers [76], including a group of 963 UN soldiers on a three-dose regime [97]. Most vaccinees who received all three doses seroconverted [97]. This vaccine was further developed into the inactivated vaccine TSI GSD 200 by the United States Army Medical Research Institute of Infectious Diseases (USAMRIID). Data from a 12-month vaccination trial using this vaccine preparation indicated the induction of long-term immunity in vaccinated humans after a primary three dose vaccination followed by a single six-month boost [68]. A formalin inactivated vaccine [73], with virus grown in BHK-21 cells, is commercially available from the Onderstepoort Veterinary Institute in South Africa for veterinary use. Also, another formalin-inactivated vaccine (lot NBR103) developed for use in humans [75], which was prepared from the 180th mouse passage of Entebbe strain of RVFV grown in monkey kidney cell culture [77], is available and has been used to control outbreaks of RVFV in South Africa. Another formalin inactivated vaccine was prepared by the Veterinary Serum and Vaccine Research Institute (VSVRI) in Egypt from a local Egyptian RVFV isolated in 1977 (ZH501) and was used in Egypt to vaccinate livestock. The virus for this vaccine was also propagated in BHK-21 cells, inactivated with 0.5% formalin, and adjuvanted with aluminum hydroxide [78]. Although inactivated vaccines induce neutralizing antibody titers, the requirement for a booster vaccination to induce a protective immune response makes their use in resource-limited settings, most notably in transhumance and pastoral communities in Africa, problematic [98]. This represents a major reason for the search for a better alternative, e.g., live attenuated vaccines.

### 3.2. Live-Attenuated RVFV Vaccines

#### 3.2.1. Smithburn Vaccine

The modified live Smithburn vaccine is one of the oldest and most widely used vaccines for controlling RVF in Africa. The vaccine virus is a neurotropic RVFV strain [66], isolated from a mosquito *Eretmapodites* spp. in Uganda in 1944 [6,99]. The vaccine was first produced as an avianized (by repeated culture in a chick embryo) live-attenuated animal vaccine in South Africa in 1951 via serial passages in a mouse brain (passage 102) and embryonated chicken egg (passage 54) [6]. Subsequently, in 1958, the virus strain that has only been passaged in a mouse brain 103 times was used as the only stock for further vaccine development. Since 1971, the vaccine stock has been propagated in BHK-21 cells for the formulation of a freeze-dried vaccine for the immunization of susceptible livestock in South Africa, many other countries in Africa, and in Saudi Arabia [6]. The same vaccine stock at passage 106 has been used in Kenya since 1960 [6,100,101] and the seed virus was used to produce a live-attenuated vaccine in Egypt in 1994 [78]. In the period between 1951 and 1986, until the time when sales records were made public, millions of Smithburn vaccine doses were sold in South Africa, Kenya [6], Zimbabwe [10], Namibia, Egypt, and Israel [6]. In recent years, a substantial amount of vaccine doses, mainly originating from South Africa, have been used in eastern Africa and Saudi Arabia [6]. The appeal of a Smithburn vaccine in African countries and Saudi Arabia for controlling RVF is attributed to its relatively low cost and its ability to induce long-lasting immunity after a single administration. The latter attribute makes the vaccine practical for administration in livestock maintained under the traditional extensive management system including livestock owned by nomadic and pastoralist communities.

However, there are risks associated with the use of the Smithburn vaccine in endemic settings in Africa. Genetic reassortment among RVFV isolates in nature [102,103,104] presents a major risk factor. Decades of widespread use of the Smithburn vaccine in Africa resulted in a significant genetic footprint of the vaccine virus across geographic locations in different countries in Africa. Indeed, molecular epidemiology studies by Grobbelaar et al. [6] demonstrated a substantial genetic footprint of the vaccine isolate in several lineages consisting of virus isolates originating in Southern, Central, Eastern, and Northern (Egypt) Africa including a strain, SA184/10, isolated from a human patient who was potentially co-infected with a wildtype virus and the Smithburn vaccine strain. In Eastern and Southern Africa, large scale animal vaccination campaigns were often only carried out during active disease outbreaks [6]; and livestock vaccines for mass vaccinations are typically sold in multidose vials and administered with automatic syringes with intermittent changes of needles. In such situations, the vaccine is likely to be administered to some animals that are already infected and viremic, thus facilitating the serial transfer of a wildtype virus. Such vaccination campaigns. as well as the uptake and transmission of the virus by vectors, might have resulted in co-infections with the vaccine and wildtype virus, creating a generation of recombinant and reassortant genotypes [6,105]. In addition, vaccinations with Smithburn have been associated with abortions in pregnant livestock and teratogenic effects in fetuses [78], and like most live attenuated vaccines, they carry the risk of reversion to virulence, presenting the urgent need for developing safer and efficacious alternatives. 

#### 3.2.2. MP-12 Vaccine

The MP-12 vaccine was promoted as an alternative to the RVF Smithburn vaccine. Development of this vaccine was undertaken by USAMRIID with the aim of producing a vaccine for both human and veterinary use. MP-12 was developed from the virulent ZH-548 strain following 12 passages in MRC-5 cells in the presence of a chemical mutagen, 5-fluorouracil, resulting in redundant mutations in all three genomic segments [79,80]. To evaluate its potential as a human vaccine, the vaccine was tested in Rhesus Macaques via mucosal (oral, intranasal drops, or small particle aerosol drops) and parenteral immunizations [81,106]. All vaccinated monkeys developed neutralizing antibody titers of ≥1:40 that were protective against the virulent RVFV ZH501 challenge [81]. Further clinical testing in more than 100 human volunteers showed that the vaccine is well tolerated and immunogenic when an adequate dose is administered [107]. MP-12 has also been extensively evaluated as a livestock vaccine. Due to concerns about its potential to cause abortions and/or teratogenicity when administered to pregnant animals, the vaccine was evaluated in a sheep pregnancy model. The immunization of ewes at 70–100 days of gestation with MP-12 induced neutralizing antibodies (1:80 to 1:320) did not result in fetal abnormalities and abortions [108,109]. Newborn lambs from the immunized ewes acquired neutralizing antibodies via colostrum [110] indicating a potential beneficial effect of immunizing pregnant dams to elicit protection in newborn animals via the passive transfer of maternal antibodies.

The MP12 vaccine was shown to be safe and immunogenic in newborn two-day-old to three-month-old lambs by inducing neutralizing antibodies against RVFV following vaccinations [108,111,112]. Furthermore, Morrill et al. [113] reported that the vaccine did not induce abortion in pregnant cows or shedding of the vaccine virus in lactating cows. However, the MP12 vaccination of ewes in early pregnancy at the 28th day of gestation induced abortion in 4% of ewes and teratogenic effects in 14% of newborn lambs [114], suggesting that the MP12 live vaccine retains some residual virulence and could cause abortions and teratogenic effects in pregnant ewes. Although there are no reports of viremia occurring in MP-12-vaccinated livestock alleviating fears of potential assortment between the vaccine strain and wildtype virus in endemic settings, the functionality of the NSs gene [115] raises legitimate concerns about the safety of the vaccine in pregnant animals [40]. The NSs protein is a major virulence factor capable of downregulating host innate antiviral response factors, including protein kinase R [21,115]. Additionally, NSs forms filamentous structures in the nuclei of infected cells [116,117] interacting with constitutive heterochromatin clusters of pericentromeric DNA sequences in host cells [118], and with the sin3A-associated protein 30 (SAP30) [19]. This enhances the interaction with heterochromatin leading to chromosome cohesion and segregation effects, and could help explain the mechanism by which RVFV causes abortions and fetal deformities in infected animals [40]; and given the virulence of the NSs gene in MP-12, it may further explain the abortions and teratogenic effects of the MP-12 live vaccine.

## 4. Novel RVF Vaccines

### 4.1. Type I Vaccines

#### 4.1.1. Recombinant Protein Vaccines

Recombinant protein vaccines are based on antigens generated by gene cloning to produce large quantities of purified or unpurified antigens. For RVFV, the targets for recombinant protein vaccine development have been the viral structural proteins, the glycoproteins Gn and/or Gc [28,29,45,46,56], or the nucleocapsid protein [56,119,120]. These vaccines have been evaluated in various animal models (Table 1). In a recent study in a ruminant model in sheep/lambs, a recombinant subunit vaccine formulation consisting of RVFV glycoproteins Gn/Gc expressed in the baculovirus system showed a strong neutralizing antibody response and conferred complete protection against virulent challenge [27,28]. Production of the viral glycoproteins Gn and Gc in glycosylated forms, a posttranslational modification shown to enhance antigenicity for various antigens [121], was most likely a key factor. Glycosylation was facilitated by the addition of a signal peptide sequence to the structural glycoproteins ensuring processing of the proteins through the cellular glycosylation pathway [122]. Vaccination of lambs with a Gn protein only expressed in a eukaryotic (insect cells) expression system also conferred protection against virulent challenge [29].

The RVFV nucleoprotein (N) has also been considered a potential candidate for subunit vaccine development. Although the N is the most abundant and highly immunogenic viral protein, the immunization of mice using nucleoprotein-based vaccines conferred only partial immune protection [56]. Remarkably, none of the mice vaccinated with the purified N protein seroconverted, but 60% of the vaccinates survived virulent challenge, suggesting the contribution of other factors, most likely cellular immunity, to protective immunity against RVFV. In a recent study, the functional role of the RVFV N protein as a potent human T cell immunogen capable of eliciting broad, immunodominant CD8^+^ T cell responses has been demonstrated [119]. In general, the lack of optimal protection conferred by N protein vaccinations described in several studies could be attributed to the induction of non-neutralizing antibodies. However, vaccinations with purified and crude RVFV surface glycoproteins have been shown to induce strong neutralizing antibody responses that conferred complete or a high level of protection against virulent RVFV challenge. A major advantage of using the glycoprotein-based subunit vaccines is their high safety profile that allows production and use in non-endemic areas; and the absence of the viral nucleoprotein as a constituent in the vaccine formulation allows the development of a DIVA vaccine using N-based serological assays as companion diagnostic tests. Although there appears to be a role for the N protein in enhancing or eliciting some level of protection, these reports suggest that RVFV surface glycoproteins represent the major targets for neutralizing and the protective immune response.

#### 4.1.2. DNA Vaccines

One of the developments in the RVF vaccine field in recent years involves the use of DNA vaccines encoding viral structural antigens for immunization. RVFV DNA vaccines have been tested in animal models with mixed results in terms of efficacy or performance, including gene sequences of the surface glycoproteins Gn and/or Gc, as well as nucleoprotein N. Spik et al. [48] assessed the immunogenicity of a combination DNA vaccine consisting of plasmid constructs encoding Gn and Gc of RVFV, Crimean-Congo hemorrhagic fever virus (CCHFV), Hantaan virus, and preM and E genes of tick-borne encephalitis virus (TBEV). The RVFV and TBEV DNA vaccines elicited antibodies and protected mice from virulent challenge. In contrast, mice vaccinated with a DNA vaccine expressing Gn and Gc failed to seroconvert with only a 20% survival rate [56]. To enhance vaccine immunogenicity and efficacy, mice were subsequently primed with a DNA vaccine and then boosted with a lumpy skin disease virus-vectored recombinant vaccine expressing the RVFV glycoproteins Gn and Gc; this increased protection to a 40% survival rate [56]. The immunization of mice with naked/plain cDNA encoding N and Gn/Gc of RVFV induced a strong immune response. Vaccinations using cDNA encoding N protein induced a strong humoral and lymphocyte proliferative response, whereas vaccinations with cDNA encoding the glycoproteins induced a strong neutralizing antibody response. Although none of the mice died after the challenge, only 50% and 63%, respectively, of the mice were protected from clinical signs of the disease [47]. The vaccination of type I interferon deficient-mice (IFNAR−/−) with plasmid DNA encoding RVFV N induced only partial protection upon challenge with the MP12 virus, whereas vaccinations with DNA encoding the viral glycoproteins Gn/Gc elicited a dose-dependent protection, which was attributed to the induction of neutralizing antibodies [123]. To augment the protective efficacy of N-based vaccines to the level observed for the glycoprotein constructs, Boshra et al. [124] vaccinated IFNAR−/− mice with a DNA construct encoding ubiquitinated form of the RVFV N protein, resulting in a significant increase in the survival rate. The vaccinated mice displayed higher levels of non-neutralizing anti-N antibodies and antigen-specific T-cell responses suggesting a role for cell-mediated responses in protection against RVFV. Overall, the relative theromostability and low cost of DNA vaccines have made the platform attractive for the development of vaccines for use in the tropics/developing countries, where the maintenance of a cold chain, especially in rural areas, may be problematic. However, their use is limited by their inability to induce strong and protective host immune responses indicating that more research into improving DNA vaccine immunogenicity is needed for the use of this vaccine platform as an alternative and sustainable RVF disease control strategy.

#### 4.1.3. Virus-Like Particles

A major advance in RVFV vaccine development is the assembly of structural RVFV proteins in eukaryotic expression systems to produce what is called virus-like particles (VLPs) [125]. VLPs mimic the overall structure of the virion without containing infectious genetic material. They have the authentic conformation of viral capsids or surface proteins seen with attenuated virus vaccines, without any of the risks associated with virus replication and inactivation. RVFV VLPs were made from the viral nucleoprotein [126] and the glycoproteins [89], or both [61]. Studies on the immunogenicity and efficacy of the vaccine constructs composed of structural glycoproteins demonstrated the induction of neutralizing antibodies and protective immunity against virulent RVFV challenge in mice [46,49]. The advantage of VLPs as a viable vaccine platform is the ability to be taken up into antigen presenting cells and stimulate both MHC class I and class II responses [127], as well as cellular and humoral immune responses [128,129,130,131]. Further testing to determine the efficacy of VLPs as a suitable vaccine candidate in ruminants is required (Table 1). Despite progress in VLP vaccine development, the adoption of VLPs as an RVF vaccine platform for livestock is yet to achieve success, primarily due to costs associated with mass scale production. 

### 4.2. Type II Vaccines

#### 4.2.1. Clone 13

A naturally attenuated mutant designated Clone 13 is a plaque-derived clone that was isolated in the Central African Republic from a patient with clinical RVF. The isolate had a natural deletion of 70% of its NSs gene, and was attenuated in mice [62]. Several studies that evaluated Clone 13 have shown that the vaccine was highly immunogenic and did not cause abortion or teratogeny upon vaccinating pregnant ewes [10,63,132]. Another study in calves demonstrated the vaccine was efficacious against heterologous virulent challenge using the RVFV M35/74 strain [82]. An additional field evaluation of the vaccine performed in West Africa demonstrated that the vaccine is well tolerated in West African breeds of sheep and goats, including pregnant animals [133]. Furthermore, the Clone 13 virus did not cause detectable viremia in vaccinated ruminants, thus minimizing the risk of vaccine virus transmission to the fetus or to mosquito vectors [63]. However, a recent report indicated that Clone 13 could cross the ovine placental barrier and be associated with fetal infections, malformations, and stillbirths when administered in an overdose to pregnant ewes in their first trimester [134]. It must be noted that the overwhelming consensus appears to be that Clone 13 is safe in sheep and cattle regardless of physiological status when the recommended (not overdose) dose of the vaccine is administered. There has been a major step in Africa towards the widespread adoption of Clone 13 for RVF control. The Clone 13 vaccine has been licensed in South Africa since 2010, with more than 19 million doses already used in the field [135], as well as in Kenya for commercial use. The vaccine is also registered in Botswana and Namibia [135]; and a recently concluded safety trial in Senegal [133] could facilitate the registration and commercialization of the vaccine in West Africa.

Recently, a thermostable clonal isolate of Clone 13, named CL13T, has been described [83]. The vaccine candidate was immunogenic in sheep, cattle, and goats [83], and safe and immunogenic in pregnant camels [84]. Further studies are required to demonstrate the efficacy of this novel vaccine.

#### 4.2.2. R566

Another live-attenuated vaccine construct R566 was created via the reassortment of Clone 13 virus with MP12 through co-infection in Vero cells [85]. The R566 virus combines the S segment of Clone 13 virus with attenuating mutations on the M and L segments of MP12 virus. Two of eight lambs vaccinated with the R566 vaccine construct developed mild fever after challenge, which was associated with low levels of viral RNAemia [85]. The induction of partial protection suggests that further improvement of this vaccine or vaccination method is required.

#### 4.2.3. Genetically-Modified MP-12 (Deletion Mutants)

To improve the safety of MP-12, reverse genetic techniques were used to introduce deletions and/or insertions in the S and M RNA segments mainly targeting genes encoding the two non-structural proteins, NSs and NSm proteins, respectively [22,65,86,136]. Morrill et al. [65] evaluated the safety and immunogenicity of two MP-12 deletion mutants, NSs (arMP-12ΔNSs16/198) and NSm (arMP-12ΔNSm21/384), in pregnant ewes, with the NSs deletion mutant showing a lower immune response compared to the NSm mutant, thus making the latter vaccine candidate a more attractive choice for human and livestock use. No abortions occurred, though a single fetal death in each of the arMP-12 and RVF MP-12 groups, which could not be attributed to the vaccines, was found at necropsy. Although the overall results of these experiments look promising, further studies are required to demonstrate the clear safety profiles and efficacy of these vaccines.

#### 4.2.4. Genetically-Modified ZH501 (Deletion Mutants)

To produce a live-attenuated RVFV vaccine with a good safety profile, Bird et al. created a recombinant double deletion mutant that lacked NSm and NSs genes [23,59]. The recombinant virus was rescued from sequences of the RVFV ZH501 strain. Experiments using a lethal disease model in rats demonstrated that the double deletion mutant was attenuated and after challenge, no detectable viremia or clinical illness was observed [23]. The vaccine was further tested for safety, immunogenicity, and efficacy in sheep, including pregnant ewes at different days of gestation [59]. Administered at doses ranging from 10^3^ to 10^5^ PFU, the vaccine was safe and immunogenic for adult sheep, and protected pregnant ewes from abortions or fetal malformations. The double-deletion NSs/NSm mutant vaccine induced sterilizing immunity post challenge in the vaccinated animals and was DIVA-compatible [59]. However, live-attenuated concerns about the safety of live attenuated vaccine usage in non-endemic countries remain.

#### 4.2.5. Four Segmented RVFV

Recently, a four-segmented RVFV strain was developed [87] and evaluated for efficacy in lambs [88]. The vaccine construct was developed by splitting the M-genome segment and the deletion of the NSs gene. This virus was attenuated, safe for pregnant ewes [137], and the vaccination of lambs via the subcutaneous (10^5^ TCID50) or the intramuscular (10^6^ TCID50) route prevented them from clinical signs and induced sterile immunity [88]. The vaccine is yet to be evaluated in other animal species such as cattle.

Overall, a major obstacle to the adoption of modified-live viruses as a vaccine platform for RVF is resistance in certain countries and continents to the use of genetically modified organisms, particularly in humans and livestock entering the food chain, and the risk of reverting to virulence, as shown for attenuated herpesviruses [138]. Further testing and a broad-base evaluation of these vaccines, as well as public education or increasing awareness, are required before they gain widespread acceptance in both developing and developed countries.

### 4.3. Type III Vaccines 

#### Virus-Vectored Vaccines

Several virus vectors have been created as vaccine candidates for RVFV. These vectors expressed the RVFV Gn and/or Gc glycoproteins and non-structural proteins and have been tested in mice and sheep. Heterologous vectors include a lumpy skin disease virus (LSDV) (family Poxviridae) designed to protect against RVFV and lumpy skin disease [56,90]; and monovalent constructs such as Newcastle disease virus (NDV) [92,93], an alpha virus (Sindbis) replicon [55], chimpanzee adenovirus construct (ChAdOx1-GnGc) [57,58], modified vaccinia Ankara (MVA) and recombinant vaccinia virus Copenhagen strain (vCO) [91,94], and an equine herpesvirus type 1 (EHV-1) vector [95] for protection against RVFV. Also, non-replicating RVFV replicons have been created [52,53,54]. Sheep vaccinated with a recombinant LSDV vaccine were significantly protected against RVFV and sheep pox virus [90]; and the NDV-based vaccine elicited RVFV neutralizing antibodies in both calves and lambs, including protection of the latter from viremia, pyrexia, and mortality [92,93]. Vaccinations with alpha virus replicons conferred 100% protection against lethal RVFV challenge in mice, and elicited RVFV-specific neutralizing antibody responses in vaccinated sheep [55]. Warimwe et al. showed that a single-dose immunization with ChAdOx1-GnGc provided complete protection against RVFV challenge in sheep, goats, and cattle [57]. The vaccination of lambs with an MVA vectored RVF vaccine conferred only partial protection and did not provide sterile immunity nor protection from clinical disease [94]; whereas vaccination with vCo constructs conferred partial protection in mice and elicited neutralizing antibody titers in baboons [37]. The efficacy of the vCo vaccine constructs is yet to be evaluated in a non-human primate or ruminant model. The efficacy of the recombinant EHV-1 vector has not been evaluated either; however, immunogenicity studies demonstrated the induction of RVFV neutralizing antibody titers in sheep [95]. Vaccinations with non-spreading single cycle RVFV replicons induced 100% protection in mice [52] and provided complete protection and sterile immunity in lambs [54]. Although live virus vectors have advantages as veterinary vaccines in resource-limited settings by inducing long-duration immunity, they may have problems with homologous booster vaccinations due to anti-vector immunity [39], as well as potential pre-existing anti-vector immunity. For example, the endemicity and high prevalence of LSDV in most of sub-Saharan Africa suggest the existence of widespread pre-existing immunity in local cattle populations, which may negatively affect the efficacy of the recombinant LSDV vaccine for RVFV in endemic settings. Therefore, alternative approaches such as NDV-based vectors, where the target species for RVFV-sheep and cattle are not natural hosts of NDV, are advantageous because they are unlikely to be compromised by preexisting immunity in field-exposed animals.

## 5. Perspectives on RVF Vaccines, Vaccination Policy and Regulation

While RVF is recognized as an important zoonotic disease threat of global importance, there is little information available on the public health and socioeconomic benefits of livestock vaccinations in endemic countries. Additionally, there is a lack of comprehensive studies on the return on investment in controlling RVFV via routine or strategic livestock vaccinations in endemic countries. Given the infrequent nature of explosive or overt RVF outbreaks in humans, it has proven even more difficult to attract the necessary policy, political, or donor support for routine or strategic RVF vaccinations for livestock. Reliable figures on the economic impact of RVF in sub-Saharan Africa are unavailable. However, the 2000 outbreak in East Africa and Saudi Arabia resulted in stopping the importation of live animals from the Horn of Africa (Ethiopia, Somalia, and Kenya) to the Arabian Peninsula. This trade inflicted significant economic damage, with an estimated loss of US $132 million in Somalia and Kenya including a 42% decline in the overall productivity of the region [139].

In the last decade, the frequency of RVF outbreaks in sub-Saharan Africa appears to have increased quite dramatically. In 2006, an outbreak occurred in Kenya, Tanzania, and Somalia. This outbreak was confirmed by the isolation of the virus from 10 patients in Kenya [12,140,141]. In 2010, an outbreak of the disease in South Africa resulted in 172 human cases resulting in 15 deaths. Occupational data of 139 of the 172 individuals affected indicated that 81% of them had direct contact with animals. Again, in South Africa and Namibia, following sporadic outbreaks of the disease in 2008–2009, a major outbreak occurred in 2011 resulting in 250 human cases including 25 deaths, and more than 14,000 animal cases, with 8000 deaths. Also, occupational data indicated that most patients had a history of direct contact with infected animals [142,143]. In October 2012, an outbreak of RVF occurred in Mauritania [4]. A large but unspecified number of small ruminants were affected, together with 34 confirmed human cases, including 17 deaths (a case fatality rate of 50%) [11]. In a cross-sectional study of miscarriages of Sudanese women, 28 pregnant women with febrile illness were found to be infected with RVFV, of which 54% suffered a miscarriage [144]. Here again, most of the human cases had a history of contact with animals. From September 2013 to January 2014, cases of RVF were reported in camels and small ruminants in Mauritania and Senegal, and epidemiological surveillance also detected human cases in several regions in Senegal [5,145]. In Mauritania in 2015, 31 patients who presented with severe clinical symptoms of RVF tested positive for RVFV and the outbreak resulted in eight deaths [146]. In March 2016, an RVF outbreak occurred in the Kabale District in Western Uganda in East Africa. The incident started with a butcher who reported to a local hospital with RVF disease symptoms and subsequently tested positive for RVFV [147]. In Niger, West Africa, in 2016, a RVF disease outbreak resulted in 348 human cases, with 33 deaths. Although data on livestock morbidity and mortality was unavailable, animal movements, trade, and changes in weather conditions were considered the main risk factors for disease spread and transmission [148].

The recent RVF outbreaks seem to indicate that human cases of RVF are a consequence of contact or association with infected livestock. Livestock are highly susceptible to RVFV infection and provide the key ecological link between the primary vector, the *Aedes*/*Culex* spp., and the human population [149]. It is therefore justified to recommend the routine vaccination of susceptible livestock in endemic countries as an important strategy to prevent human disease, socioeconomic loss, and disease outbreaks. Unfortunately, there is currently no policy in place in support of the targeted or routine vaccination of livestock in any of the countries endemic for RVF throughout sub-Saharan Africa. Vaccination campaigns are mainly reactive in response to disease outbreaks mostly involving human casualties, making the impact of these interventions less effective. The approach of relying on early warning systems based on satellite remote sensing data has proven successful in the past in predicting RVF outbreaks in Kenya [150,151]. However, the success of this approach, which relies heavily on climate and rainfall data in predicting future RVF outbreaks, is not guaranteed to be consistently accurate in the future. Moreover, the cost of the technology is another important consideration. The strategic vaccination of livestock during interepidemic periods or as a preemptive countermeasure could eliminate one of the main sources of human infection and limit the scope of epidemics or disease outbreaks [149]. We argue that this approach represents the most sustainable solution applicable to endemic countries that could help disrupt disease transmission and prevent the further spread of RVFV to non-endemic countries. Economics remain a key determinant for RVF vaccine development. The infrequent nature of RVF disease outbreaks has diminished the incentive for manufacturing companies to produce and stockpile vaccines that have a relatively short shelf life, or for endemic countries to utilize the limited available resources to subsidize routine vaccination programs. Taking these concerns into consideration, it is reasonable to suggest that the implementation of a successful program of routine livestock vaccination for RVF in endemic countries will require sustained commitment in the endemic countries, as well as material and financial support from Western donor countries or multilateral organizations such as WHO, FAO, and OIE, etc. To gain political support, research that demonstrates the long-term benefits of routine RVF vaccinations of livestock for public health, economic benefits, and threat reduction is critical.

Several RVF vaccines have been developed by different research groups (Table 1). Vaccination policies and the use of vaccine type may differ in endemic and non-endemic countries depending on the major objective to be accomplished. In non-endemic countries, where control and eradication is a major objective in the face of an outbreak, the use of vaccines that allow for DIVA represents the best option (Table 2). Also, some countries in Africa, such as Botswana, which rely on the export of livestock or livestock products to non-endemic countries, e.g., in Europe, will also pursue a vaccination policy geared towards eradication. In such instances, a DIVA vaccine is required and the application of companion diagnostic tests will allow compliance with international trade restrictions that are often imposed during active RVFV outbreaks, further worsening the negative economic consequences of the disease. In endemic countries, the choice of which vaccine candidate could be used to control RVF is more flexible. The use of DIVA vaccines is comparatively less important, although may be a better option if eradication is the goal. However, the use of non-DIVA compatible vaccines as a routine control measure and to control disease outbreaks remains a viable option. Indeed, inactivated and live-attenuated (Smithburn) vaccines have been used in Africa for decades; however, the requirement for repeated vaccinations (for inactivated vaccines) and risk for teratogenic effects, abortion, and potential reassortment/reversion (for the Smithburn vaccine) means that the research on the development of new generation efficacious vaccines with a high safety profile is still necessary. Another important vaccine attribute that may differ in terms of importance for developing and developed countries is thermostability. In the tropics, most particularly in sub-Saharan Africa, where the maintenance of cold-chain in rural areas may be problematic, the thermostability of vaccines represents an important attribute for vaccine development (Table 2). The infrequent nature of RVF outbreaks suggests that the vaccine price could be the single most important constraint to livestock producers in developing countries to adopt routine or strategic livestock vaccination. This suggests that any suitable vaccine must be sold at a price that is affordable, most likely via subsidies provided by the respective target countries, as well as support from donor countries and multilateral organizations.

## 6. Conclusions

Ultimately, the prevention of catastrophic outbreaks of RVF will require a One Health approach [152] involving concerted efforts by medical and animal health personnel. Given the stringent regulatory requirements and approval process required for human vaccines, the mass vaccination of human populations against RVF may not be a viable option at present or in the near future; and a more sustainable and rational strategy would be the use of investigational new vaccines to vaccinate at risk personnel or groups—veterinarians, slaughter house workers, laboratory and research personnel, and military and non-military personnel for deployment in RVFV risk zones. For this, vaccines lacking infectious genetic material (e.g., recombinant protein-based, VLPs) and replication-deficient virus vectored or replicon vaccines with a high safety profile could be the most appealing in getting regulatory approval for use in humans. Vaccine regulation is a complex process. The lack of adequate regulatory infrastructure in many endemic countries in sub-Saharan Africa presents significant obstacles to RVF vaccine development and disease control. Thus, strengthening regulatory authorities in endemic countries in sub-Saharan Africa, at national and/or regional levels, including an expansion of the mandate of regulatory authorities in non-endemic developed countries such as the United States (Food and Drug Administration) and Europe (European Medicines Evaluation Agency) for licensing RVF vaccines for use in developing countries, should be given serious consideration. The question as to what type of vaccine would be most suitable for use in RVF disease outbreak situations in both endemic and non-endemic countries is significantly important. Novel Type I and Type III vaccines (e.g., recombinant protein-based vaccines, VLPs and DNA vaccines, replication-deficient virus vectors, or replicons) that are DIVA-compatible with a high safety profile and pose a minimal or no environmental risk are most desirable. In the long run, strategic, targeted livestock vaccinations could be a solution for the prevention of the zoonotic spread of RVFV and funding agencies and industry should be encouraged to support such integrated approaches. As is the case for the successful eradication of brucellosis among livestock in the US [153], bluetongue in Europe/Germany [154], and rinderpest in Africa [155,156], the systematic or routine vaccination of livestock will disrupt the transmission cycle of RVFV and diminish or potentially eliminate the threat of this important disease to both humans and livestock.

## Figures and Tables

**Table 1:** Status of RVF vaccines and vaccine candidates evaluated for efficacy in different animal models.

Type of Vaccine	Host Species Evaluated/Used in					DIVA	References
	Mice	Sheep	Cattle	NHP	Other		
**Inactivated**							
NDBR103	√				√ ^a^	No	[75,76]
TSI GSD 200					√ ^a^	No	[68]
Formalin Inactivated (South Africa)		√	√			No	[73,77]
Formalin inactivated (Egypt)		√	√			No	[78]
**Genetically non-modified-live**							
Smithburn	√	√	√			No	[66,78]
MP12	√	√	√	√	√ ^a^	No	[65,79,80,81]
**Genetically modified-live**							
Clone 13, Cl13T	√	√	√		√ ^b^	Yes	[62,63,82,83,84]
R566		√				Yes	[85]
Recombinant MP12 Δ/mutants	√	√				Yes	[22,65,86]
Recombinant ZH501 Δ/mutants	√	√				Yes	[23,59]
Four-segmented RVFV	√	√				Yes	[87,88]
**Recombinant protein vaccines**	√	√	√			Yes	[28,45,46]
**DNA vaccines**	√					Yes	[47,48,56]
**Virus-like particles (VLPs)**	√					Yes	[46,49,89]
**Virus replicons**	√	√				Yes	[52,53,54]
**Virus-vectored**							
Poxviruses	√	√		√		Yes	[56,90,91]
Newcastle Disease Virus	√	√	√			Yes	[92,93]
Chimpanzee adenovirus	√	√	√		√ ^b^	Yes	[57,58]
Modified vaccinia Ankara	√	√		√		Yes	[91,94]
Equine herpesvirus virus type 1		√				Yes	[95]

a = Human volunteers; b = goats and camels; NHP = non-human primates.

**Table 2:** Relative importance of vaccine attributes in endemic and non-endemic countries.

Vaccine Attribute	Relative Importance	
	Endemic	Non-Endemic
DIVA compatibility	+/−	+++
Thermostability	+++	+
Long shelf life	++	+
Single shot	+++	++
Cost per vaccine dose	+++	+
